# Acute Articular Rheumatism

**Published:** 1875-08

**Authors:** C. H. Tidd

**Affiliations:** Middleport, Ohio


VOL. V.—AUGUST, 1875.—NO. 8.

### Original Communications.

### ACUTE ARTICULAR RHEUMATISM.

BY C. H. TIDD, M. D., MIDDLEPORT, OHIO.

*Mr. President and Gentlemen of the Society:*

Acute articular rheumatism is at once the most fickle and obstinate disease which we, as physicians, are called upon to treat. As it has always done in the past, so will it in the future continue to baffle the skill of the most experienced of the profession, so long, at least, as they treat it upon the theory that the *blood* is the fluid at fault, and lose sight of other factors, which, to my mind, are more prolific in its production than the blood; and that such is the case, I am fully convinced, and hope to be able so to elucidate my views as to make them plain to each of you; and in doing so, I shall ask you to lose sight of the blood for the present, and direct your minds to the *nervous* system, for reasons which will explain themselves to your minds, as we proceed. Acute articular rheumatism is an affection of the articulations and joints arising from some morbid condition of the system, which is usually conceded to be an excess of *lactic acid* in the *blood;* yet all will agree that this concession is upon purely hypothetical grounds, insomuch as the fact has not been established by demonstrative proof. Even assuming, for sake of argument, that the hypothesis be true, it is evident that the presnce of lactic acid in the blood in excess is an effect of morbid conditions, of which nothing positive is at present known. Lactic acid is formed by the destruction of sugar in the lungs, and also in the decomposition of gelatinous and albuminoid tissues, and this in a state of health. Now, if the presence of it in the blood was *the* cause of this affection, would we not meet it much more frequently than we do?—and would it be overcome by the various modes of treatment which are instituted, which, in many cases, seem to be diametrically opposite? I think not, but will defer giving my reasons for such belief until I come to discuss the treatment. Again, some writers claim that the disease is due to the suppression of the functions of the skin, and attribute its action to the influence of cold upon the surface of the body. This theory seems tenable, from the fact that almost every patient is apt to refer the disease to some sudden and severe exposure, and are often able to fix the date of said exposure, and claim it as the origin of their trouble. This view, in the opinion of your essayist, is without firm foundation, for how often do we meet cases of prolonged exposure to cold, in which even parts of the body are frozen, which are not followed by this disease? That the causation may involve the agency of cold, cannot be denied *in toto*, but we hope to be able to show that cold acts only as an *exciting* cause, and that the development of rheumatism is only a secondary effect of this agent. What, then, *is* the *primary* cause of the *disease?* In answering this question, I shall advance only my own views, being fully cognizant of the fact that I stand, so far as I know, almost entirely alone, with scarce a single authority to back me in my assertions, but, on the contrary, with the masses of our *teachers* and *writers* directly opposed to me; and in presenting views so diametrically opposite to *these*, as well as to your own, which I have often heard expressed by you, I can only say, in the language of our fellow, Dr. Knight: "Gentlemen, you have given me your theory without proofs to back you, and I cannot accept such arguments without farther investigation for myself, or sufficient *proof* from you that your views are correct." If the profession were a unit in all things, especially in their views of the causation and treatment of disease, there would be no need of our meeting here, nor for the many medical journals which are printed for our instruction. Having had occasion recently to watch closely and carefully a case of acute articular rheumatism, which, as you are all aware, occurred in my own family, I was led to study the case more closely than I could possibly have done in an outside patient, and I became fully convinced that the primary seat of the difficulty must be sought in the *nervous* system and not in the blood, and that whatever we may find of acid products (lactic) in the blood, are only secondary effects, and are due to the morbid condition of the nervous system, and can only be reached by those agents which exert their remedial influence upon the nerves. And if each of you will recall the cases treated, and the agents employed, I apprehend that it will not require any very strong arguments on my part to convince you that such is the case. You ask what part of the nervous system I deem most at fault.? I answer unhesitatingly, the *spinal cord* primarily, and its larger branches with their ramifications secondarily, and especially those branches connected with the brachial, sacral and sciatic plexus. I repeat, rheumatism is a *constitutional disease*, the inflammation being brought about and maintained in consequence of functional derangement of the spinal cord, which derangement is produced by either active or passive congestion, or perhaps inanition of that important appendage of the great center of the nervous system, which condition may be brought about by cold, or by any other agency that tends to break down the nervous system? If you will again recall your cases of this class, you will, I think, admit that nine-tenths of them were persons of nervo-bilious temperaments, and just that class of patients in whom every ailment is manifested by severe disorders, and a large majority of the cases will be found to be sufferers from neuralgia, nervous headaches, hepatic disorders, or some kindred or closely allied disease; and you will also remember that they were of what might be called a rheumatic diathesis, either congenital or acquired, and even when in what they termed "good health," they were scarcely ever free from aches and pains in some part of the body; in fact, were just such patients in whom we should expect to see inflammatory affections predominate. You may ask if I consider all cases of rheumatism, of whatever class, as arising from the same cause? I consider the primary cause of every case to be as stated above. Yet if you were to go still farther with your investigations, and ask why, in one case, it seems to affect the muscles alone, and in another the joints, and in still another, both muscles and joints?—I should be compelled to say, I cannot tell you, no more than I can tell you why, in one case, neuralgia affects the trifacial, and in another the great sciatic nerves. Yet that such is the case we well know. Again, it may be asked *how* does an affection of the cord produce the *inflammatory* action, and does it invariably do so? I answer, *not invariably*. Yet I think that in *nearly* every affection of the cord the calibre of the small blood vessels is diminished, and as a sequence, pain and inflammation is set up, and the patient being of a rheumatic diathesis, (even though they may never have suffered from an attack of the disease,) begets rheumatism just as they would beget other diseases if of other peculiar diathesis. But you may say these views are purely speculative, with no well authenticated facts to prove them. Granted, but allow me to ask, is not the theory as plausible as is any of the other theories which have been advanced as to the primary cause of the disease? Again, you may urge the fact that lactic acid has been found in excess in the blood of every case wherein that fluid has been tested, therefore the acid theory has, at least, that much demonstrative proof in its favor. Exactly so, but has the blood been tested in every case? And again, has not the same condition been found to exist in every form of nervous disease in which the blood has been tested with a view to this particular agent? Certainly it has. Then, if lactic acid be found in the blood of patients suffering from nervous disorders, and *rheumatism not been present*, is it logical to claim that because it is found also in this particular disease that we must admit, *nolens-volens*, that it is the prime cause of inflammatory rheumatism? Most assuredly not. Again, if we find that remedies, directly addressed to the nervous system, do in every instance remedy the difficulty, is it not fair to suppose that the nervous system is primarily at fault? I think so. And when I speak of addressing remedies directly to the nervous system, I do not mean that we *must* use *direct* nerve tonics; but our treatment, to be successful, must be with those agents that produce a revulsive or an alterative action primarily, and a tonic action secondarily; and I claim that almost the entire class of remedies usually employed does produce just this action. And this brings us to the treatment, and here let me say, I do not *condemn* the usual methods of treatment, nor do I *fully approve* of them, but do claim they are administered without, to my mind, a clear pathological understanding of the disease. That is, the remedies are addressed empirically, and while they may, and do, do as much good as though we fully understood the cause of the disease, yet we, as true physicians, are in duty bound to seek a cause for each effect. Yet I do not feel competent to instruct *you* in the treatment of your cases, and would leave this part of the field untouched did I not realize that the practice I have taken demands that I should give my views as to treatment.

The first step in the treatment is to enjoin perfect quietude; and of this it is scarcely ever necessary to assure the patient, for of all diseases where this is required, there is none wherein it is more easily obtained. The *reason* is obvious to all. And to secure this I know of no contrivance that is more applicable than the silicate of potash bandage, it being at once light and immovable, securing perfect rest to the parts, without being in the least cumbersome; but in the absence of this, any apparatus that will keep the limb perfectly quiet, and be comfortable to the patient, can be used instead. Many writers recommend opium or its salts. In the opinion of your essayist, these can only do harm, for, as is well known, opium checks the process of elimination by the natural emunctories, which is one great end to be sought. It is true it annuls the pain, but its effect is only temporary, and must be oft repeated to secure ease. Therefore we are of the opinion that it had better be substituted by chloral hydrate, or some like agent, which, while exerting a soporific effect, does not retard elimination nor interfere with digestion. Propylamin has been heralded as almost a specific in this affection, yet it has not proven itself adequate to the demands, and I apprehend that all the powers it possesses are exerted through its influence on the cord, by relieving congestion or inflammation—powers which it is known to possess.

Again, tinctura ferri chloridi has been lauded in the treatment of this disease, given in doses of from gtts. 30 to ʒss., every six to ten hours, and some writers tell us that it will relieve in from one to three days, and cure in from six to ten days any case of acute inflammatory rheumatism, yet I hardly think the experience of the profession will bear out this statement.

Veratrum viride is extensively employed by the English practitioners, given in drop doses every two hours. I do not know how they explain the *rationale* of this treatment, but the only good I should expect from its administration would be in its lessening the heart's action, thus diminishing the volume of blood that is being constantly sent to and forced through the contracted blood vessels. Considering it in this light, I should consider it a valuable adjuvant to any form of treatment.

The *ice* treatment is highly lauded by writers, yet I think that Prof. Waldee's experience and statements show us at once that the plan is fraught with danger, and should be very cautiously used, if used at all. I quote from him: "Although the various plans of water cure have familiarized the public more or less with the application of cold in febrile affections, yet very unpleasant comments may still be made as regards the use of ice in acute articular rheumatism. When sudden deaths result, as they not unfrequently have, from brain affection when it has been employed." Would we think of applying ice in a case of its twin sister, gout? Most assuredly not.

And again, others advocate just as strongly the use of hot baths, or the application of flannel cloths, wrung out of hot water, and continuously applied to the affected parts. It is a well established fact that in the treatment of neuralgic affections nothing seems to offer such speedy relief as the application of heat. This you can verify almost daily in cases of odontalgia, and in the case referred to above nothing seemed to afford such complete relief as the application of cloths wrung out of hot water and applied to the affected joints, care being taken to change them every ten or fifteen minutes. And here let me say that, after becoming fully convinced that my theory was correct, I discontinued the application to the joints, and applied the hot cloths to the entire spine, with the effect of entirely relieving the affected limb, and just as speedily as had been done by applying them directly to the limb; and I account for this action by the known powers it possesses of relaxing *muscular* fibres, by which the contraction of the blood vessels would be overcome, and the pain temporarily relieved, and I now believe that had I kept the hot cloths continually to the spine, I would have cut short the disease much sooner than was done. Surely, if the presence of lactic acid in the blood was the cause of the difficulty, heat thus apdlied would not have tended to relieve it so promptly. You may say that the diaphoresis which it produces eliminates the acid sufficiently that the pain is relieved for a time, but does it stop the manufacture of it, and would not the small quantity eliminated be almost immediately replaced by new acid? Phosphorous has also been recommended, and while I would not especially recommend the use of free phosphorous, I can conceive wherein the hypophosphites, especially in the form of the compound syrup of the hypophosphites, would prove beneficial, for here we would not only get the nerve-restoring properties of the phosphorous, but would get the eliminating properties of the potassa and soda which it contains. This treatment would certainly be acceptable to those who favor the alkaline treatment, and I will confess, gentlemen, that until within the past six months I was almost an enthusiast in this form of treatment myself, and in some cases would recommend and employ it.

Next we find a class who strongly advocate the acid treatment, and another who as strongly advocate the antacid, and each claim equally good results; and although their treatment seems to be directly opposite, I think a moment's reflection will show us, instead that they are identical, and each are working for and obtain the same results, although such opposing agents are used—each of them acting on the hypothesis that lactic acid is the cause of the trouble. Now, lactic acid in certain proportions is a normal constituent of the blood, consequently its mere presence there cannot be considered deleterious; but if the quantity be increased, then may it exert a bad influence. If it *be increased*, it must be from one of two causes; either it is not *metamorphosed* and ELIMINATED as it should be, or it is *produced* more rapidly than nature designed. If it depend on the first condition, what are the indications? Evidently to promote its expulsion, destruction, or change into those principles into which it normally resolves. To promote its expulsion is not an easy task, but to destroy it is not so difficult. Being an acid, we can easily neutralize it with an alkali; and while doing this, we are indirectly promoting its normal metamorphosis. This will be seen when we consider how this normal change is wrought. Lactic acid, after having played its part in the economy, seeks an alkali with which to combine, viz.: the free soda of the blood, and the resulting compound being oxidized, corbonate of soda and water is formed. Hence, the use of alkalies in this condition would be proper. But in cases where the *second* condition attains, what are the indications? To neutralize it? Most assuredly not, as will be readily seen when we study the forces that are at work in its production. As we before stated, lactic acid is formed from glucose, which substance is derived from a variety of sources. The change from glucose into lactic acid is a natural transition, and is formed by an alkaline condition of the blood. If the blood is already surcharged with alkalies, would we not but add fuel to the flame by administering alkalies? What, then, are we to do? The indications are plain. We should administer those remedies which will most readily change the condition, and acids, we well know, will accomplish this, and thus we see the same end is obtained by both methods of treatment. And now where are we to go for the prime origin of this difficulty, as stated above, viz.: for the cause of this increase of acid? I answer, we *must* look to the *nervous* system, for reasons which are apparent to all. Others, again, rely on colchicum largely, and I think this agent deserves more praise than many are wont to give it, as it equalizes the circulation, tones and calms the heart at once, and exerts a powerful influence on irritable nerves; besides, it is a powerful eliminator, acting freely on the kidneys, and preventing the accumulation of urea, though not so strongly marked in this latter action as is the nitrate of potassa.

Next, we notice iodide of potassium. This agent is very useful in that class of cases spoken of under the first head above, viz.: where the acid is not changed or expelled from the system as rapidly as it should be; yet I think this agent should not be used in the first stages, save in well marked cases, and then in doses of not to exceed grs. 5 every three or four hours, as it is apt to irritate the stomach; yet, in the advanced stages, I should not hesitate to give it in liberal doses, say from ℈j. to ℥ss. every four or six hours, pushing it so as to get its full influence on the nervous system as rapidly as possible; and I would, in every case, combine it with colchicum or cimicifuga, as I apprehend that either would facilitate its action. Occasionally cases will be met with that cannot tolerate it at all; it was the case in the patient previously referred to, in which the smallest dose produced a flow of urine, and a distressing diarrhœa, with great tenesums.

And next, we would notice the *insolation* treatment, and although we speak of it *last*, we do not, by any means, consider it the *least*, for, in the opinion of your essayist, we possess no more valuable adjuvant to the treatment of this disease than insolation. Of the method of conducting this it is not necessary to speak at length, as our journals for the past year or two have repeatedly detailed this. Suffice it to say, that instead of the glass tumblers usually employed, I would prefer large glass castors, which can be readily procured, and are much more elegant, while they answer every purpose; and I would call your especial attention to the necessity of keeping the bed clothes free from the floor, as well as to not allowing the clothing of the attendants to come in contact with the bed. In a few brief words, I will sum up my views as to the proper treatment in every case, and then leave the paper in your hands for discussion :

1st. I would at once (after deciding that I had a case of acute inflammatory rheumatism to deal with) have the bed insulated, for reasons which all will readily understand.

2d. I would secure perfect quietude, by means of the best appliances which I could command.

3d. I would apply cups or blisters along the spine to remove congestion, and induce normal action, as well also to render that usual sequence, peri-carditis less apt to follow.

4th. I would apply the cloths, wrung out of hot water, to the spine; also to the affected joints, not only to relax the arterial tension, but to reduce nerve energy; and when I speak of a reduction of nerve energy, I do not refer to diverting it into another channel, but to a simple decline of its *intensity*, and for this end heat is well calculated, as we all well know.

5th. I would administer such internal remedies as the case seems to demand. Phosphorous, in some form, if there seemed to be excessive nerve waste; camphor, aconite, veratrum viride, colchicum, potassa iodide, etc., if indicated to assist in the reduction of nerve energy; nitrate of potassa if indicated to relax the blood vessels, increase perspiration, or lessen the pulse, either of which it will do. And here let me add, at the risk of being considered eccentric, I do not think that nitrate of potassa should be regarded merely as an alkali, for we possess *no better promotor of the catamenia* than this agent, simply through its power of relaxing blood vessels, and its action on the cord.

6th. I would promote vasso-motor action by means of quinia, digitalis or ergot, (in small doses,) for reasons which will be so apparent that I need not explain here.

7th. I would secure ease and sleep by means of hydrate of chloral in preference to opium for two reasons:

1st. It does not derange the digestive apparatus, nor retard elimination.

2d. There is no danger of forming the opium habit, which is much to be dreaded.

Do not understand me as advocating all the above plans in any given case, unless positively demanded, but I would choose from them such as seemed to be indicated. As a prophylactic in those patients who have been subject to this disease, I would administer full doses of quinine or opium, when they are about to be subjected to undue exposure, and for these reasons: In active exercise nerve energy is being more particularly directed in certain channels—not that of inflammatory action, but rather of muscular contraction and secretion. Stimuli, therefore, besides having probably less influence upon the trophic system, are directed, (carried with the stream as it were,) and are exhausted and rendered harmless. In the administration of quinia we have the vasso-motor nerves thus pre-occupied, and also a positive reduction of trophic energy. In the use of opium, besides diminishing metamorphosis, the secretory system is stimulated to activity, and the influence of cold being in this way contracted and warded off, is much less liable to produce a recurrence of the malady.

Gentlemen, I have endeavored to give you my views of this disease as plainly as possible, feeling at the time that I was presenting views, as before stated, much at variance to your own, yet I would ask your indulgence, and request you to give these views your attention; and as proof of its correctness, would ask you to follow its teachings, so far as will enable you to judge whether the nervous system is not most at fault, as well as to decide whether or not measures addressed directly to the cord will not give you more satisfactory results than you have been wont to obtain by old methods of treatment.

